# Decoding Stimulus–Response Representations and Their Stability Using EEG-Based Multivariate Pattern Analysis

**DOI:** 10.1093/texcom/tgaa016

**Published:** 2020-05-07

**Authors:** Adam Takacs, Moritz Mückschel, Veit Roessner, Christian Beste

**Affiliations:** Cognitive Neurophysiology, Department of Child and Adolescent Psychiatry, Faculty of Medicine, TU Dresden, Dresden D-01309, Germany

**Keywords:** action control, EEG, event files, multivariate pattern analysis, signal decomposition, theory of event coding

## Abstract

Goal-directed actions require proper associations between stimuli and response. This has been delineated by cognitive theory, for example, in the theory of event coding framework, which proposes that event files represent such bindings. Yet, how such event file representations are coded on a neurophysiological level is unknown. We close this gap combining temporal electroencephalography (EEG) signal decomposition methods and multivariate pattern analysis (MVPA). We show that undecomposed neurophysiological data is unsuitable to decode event file representations because different aspects of information coded in the neurophysiological signal reveal distinct and partly opposed dynamics in the representational content. This is confirmed by applying MVPA to temporal decomposed EEG data. After intermixed aspects of information in the EEG during response selection have been separated, a reliable examination of the event file’s representational content and its temporal stability was possible. We show that representations of stimulus–response bindings are activated and decay in a gradual manner and that event file representations resemble distributed neural activity. Especially representations of stimulus–response bindings, as well as stimulus-related representations, are coded and reveal temporal stability. Purely motor-related representations are not found in neurophysiological signals during event coding.

## Introduction

Response selection in an ever-changing environment requires flexible representations of perceived objects, responses, and their interrelations. One prominent theoretical framework, the theory of event coding (TEC) (Hommel 1998, 2009, 2019; Hommel et al. 2001a), provides a unifying perspective of how perceptions, actions, and the translational processes between them are represented in the mental architecture. The TEC proposes a “common coding” mechanism (Hommel et al. 2001a) for perceived objects (external events) and motor responses (internal events) and strongly focuses on how these different aspects are being represented in the cognitive system. Perceived stimuli are represented by available features, such as color or shape, and stored in “object files” (Treisman and Kahneman 1984; Treisman 1996). Similarly, responses are represented by features, such as effector or force, and stored in “action files.” “Event files” are results of binding between object features and action features (Hommel 1998), that is, an event file is a network of stimulus–response (S–R) associations (Hommel 2011). In this network, retrieval of feature representations leads to a spreading activation in a pattern completing fashion (Hommel 2011). The main theoretical propositions of TEC have repeatedly been demonstrated at a behavioral level by 2 phenomena: the partial repetition benefit effect and the partial repetition cost effect (Hommel 2004; Colzato et al. 2006b). The first effect refers to response facilitation whenever 2 consecutive stimuli have a high level of feature overlap, and the previously associated response needs to be activated again. However, if the response needs to be changed, the S–R link needs to be reconfigured, which then leads to slower and less accurate responses—the partial repetition cost. A series of behavioral studies enriched our knowledge of how these effects work and how they are related to other cognitive mechanisms (for a review, see Hommel 2019). Importantly, feature binding and retrieval of the bound associations are identified as key processes of human action control (Frings et al. 2020). Thus, understanding the mechanisms behind the development and stability of event files is necessary for a cognitive neuroscientific approach of selecting and regulating actions. However, the neurophysiological properties of event files are still not understood. Previous research using established methods of fMRI/PET, electroencephalography (EEG), and brain stimulation identified a network behind event file coding (Elsner et al. 2002; Kühn et al. 2011; Petruo et al. 2016; Zmigrod et al. 2016; Pastötter and Frings 2018; Chmielewski and Beste 2019; Dignath et al. 2019; Takacs et al. 2020). These studies draw a picture according to which inferior parietal areas, supplementary motor areas, the dorsolateral prefrontal cortex, and the hippocampus play important roles in event file coding. Furthermore, event-related potential (ERP) methods have been used to examine the time course of different cognitive subprocesses involved during event file coding and which functional neuroanatomical structures are associated with these processes (Petruo et al. 2016; Opitz et al. 2020; Takacs et al. 2020). However, crucially, these methods could not tap into the representational content of the event files. Yet, the central element of the TEC is how S–R links are represented in the cognitive system (Hommel et al. 2001a; Hommel 2019). Essentially, it is the strength of representations that is important to consider for behavioral signatures of event coding (i.e., partial repetition costs and benefits). In the current study, we aim to fill this gap by investigating the time course and stability of the representational content of event files based on EEG data.

Some methods are suitable to answer questions related to the content and the stability of a mental representation (Haxby et al. 2001; King and Dehaene 2014; Grootswagers et al. 2016, 2017; Fahrenfort et al. 2018; Carlson et al. 2019). Particularly, multivariate pattern analysis (MVPA, also previously known as multi-voxel pattern analysis) is a tool to decode the representational difference between experimental conditions based on the observed neural patterns (Fahrenfort et al. 2018; Carlson et al. 2019). This approach goes beyond the univariate methods of previous ERP analyses of event file coding (Kleimaker et al. 2020; Opitz et al. 2020; Takacs et al. 2020). Specifically, in a univariate analysis, a priori selection of an electrode or a set of electrode is necessary, while in MVPA this subjective bias of the researcher can be eliminated by training the classifier on all channels (Fahrenfort et al. 2018). Moreover, ERPs represent a comparison between conditions based on averaged segments of EEG data. In MVPA, the segmented data, which can be either raw or decomposed, is analyzed; therefore, it can identify changes in the neural signal which does not occur in a focal manner (i.e., limited to an electrode site). One type of MVPA method is the representational similarity analysis (Fahrenfort et al. 2018; Kikumoto and Mayr 2019). This has typically been used to find correspondence between physiological and anatomical constructs. In a recent study (Kikumoto and Mayr 2019), representational similarity analysis was used to decode rule-, response-, and stimulus-related representations based on time–frequency decomposed EEG data. This analysis yielded cascadic pattern of action representations (rule, stimulus, then response) and provided evidence for a conjunctive representation of stimulus and response. However, the study did not examine the temporal stability of such representations, which is a major strength of MVPA temporal generalization procedures (King and Dehaene 2014; Fahrenfort et al. 2018). Temporal generalization is a metric of the stability of mental representations over time (King and Dehaene 2014; Grootswagers et al. 2016; Fahrenfort et al. 2018). That is, it tells when and for how long the decoded information was present in the neural activity pattern (King and Dehaene 2014). MVPA applied on EEG data has been shown to be successful to decode stimulus features, perceptual decisions, and higher-order processes, such as conceptual and semantic categories (King and Dehaene 2014). Therefore, temporal generalization is a potential tool to decode the emergence and stability of event file representations.

However, regarding the ability to decode the stability of the representational content in neurophysiological signals using MVPA, it is important to consider that EEG signals reflect a mixture of different sources (Nunez et al. 1997; Huster et al. 2015; Stock et al. 2017). Particularly during response selection, different aspects of information are intermixed in the neurophysiological signal (Folstein and Van Petten 2008) and are processed in parallel in overlapping brain regions (Mückschel et al. 2017a). This is especially the case for aspects related to perceptual processing (stimulus codes) and response selection processes (response selection codes) (Wolff et al. 2017; Mückschel et al. 2017a; Adelhöfer et al. 2018, 2019; Chmielewski et al. 2018). Thus, intermingled coding levels in the neurophysiological signal are of particular relevance during event file coding since event files establish an association/binding between these aspects. Just recently, it was shown that standard EEG/ERP data might be too “contaminated” by this mixture of signals to reliably capture correlates of event file coding (Opitz et al. 2020; Takacs et al. 2020). Specifically, event file binding effects were only detectable after applying the residue iteration decomposition (RIDE) method (Ouyang et al. 2015a). This method allows dissociating 3 clusters of activity in the neurophysiological signal: a perception-related S-(stimulus) cluster, a R-(reaction) cluster reflecting motor execution processes, and a response selection-related C- (central) cluster (Ouyang et al. 2017). RIDE postulates that different cognitive subprocesses and associated neurophysiological changes are present in single trials in parallel (Ouyang et al. 2011). Specifically, early subprocesses, such as stimulus feature integration or allocating spatial attention, are more likely locked to stimulus onset, while response preparation and evaluation are locked to responses. However, other subprocesses and their linked neurophysiological markers are highly variable in latency; therefore, analyzing stimulus-locked EEG trials would inevitably lead to a smear of components. This problem could be potentially tackled with “de-noising” single-trial ERPs or differentiating between mixed sources by using spatial decomposition methods (Ouyang et al. 2015b, 2017). Another suggested solution is using the temporal variability of single-EEG trials to create decomposed components or clusters (Ouyang et al. 2011, 2017). RIDE combines stimulus- and response-related time markers and estimated latency information to extract clusters which are either marker-locked or non–marker-locked (Ouyang et al. 2015a). Specifically, it separates a component cluster locked to the stimulus marker (S-cluster) and a component cluster locked to the response time (R-cluster). Moreover, it assumes a component with a jittered latency which is neither locked to stimulus nor to response markers (C-cluster). This latter one is detected by template matching, that is, after an initial estimation of the latency of C-cluster in single trials, S- and R-clusters are decomposed based on this estimation alongside with the information of stimulus and response markers. Then, the separated C-cluster is used as a template to re-estimate its latency. To obtain the final clusters, these steps are iterated until convergence. Importantly, the RIDE clusters have been validated by having high consistency in split-half analyses and being distinguishable from noise in terms of time–frequency pattern (Ouyang et al. 2017). The identified RIDE components are spatiotemporal, continuous waveforms (Ouyang et al. 2017), which can be further processed by either traditional univariate methods (e.g., ERPs, wavelet analysis) or potentially with multivariate approaches. In case of event file coding, applying temporal decomposition before any classification attempt can be crucial: in recent studies, event file binding effects explaining behavioral processes were either solely evident in the C-cluster data (Kleimaker et al. 2020; Opitz et al. 2020) or the effect was larger in the C-cluster than in the undecomposed EEG (Takacs et al. 2020). This underlines that event files reflect cognitive processes of S–R translation on a neurophysiological level (Verleger et al. 2014; Opitz et al. 2020; Takacs et al. 2020). Furthermore, these results strengthened the view that event files work independently of motor programs, as indicated by a lack of binding effects in the R-cluster (Opitz et al. 2020; Takacs et al. 2020).

Thus, it is very likely that only the temporal signal decomposition can provide clarity on the neural underpinnings of event files with a strong emphasis on the C-cluster activity (Verleger et al. 2014; Mückschel et al. 2017a; Opitz et al. 2020; Takacs et al. 2020). Therefore, a combination of RIDE decomposition and MVPA will provide insights into the representational content of and its stability in event files. Although MVPA has been traditionally applied to undecomposed time series EEG data, it is, in theory, applicable for a variety of domains, including time–frequency, connectivity, and decomposed EEG (Carlson et al. 2019; Kikumoto and Mayr 2019). Especially in the context of event file processes, the combination of methods is theoretically meaningful and goes beyond previous attempts (Kikumoto and Mayr 2019), since the concatenation of the temporal decomposition approach (i.e., RIDE) with temporal generalization MVPA provides insights whether specific aspects of information being processed (stimulus-related, motor response-related or processes linking stimulus evaluation and responding) show distinct temporal generalization profiles (cf., dissociation of effects in the S-, R-, and C-clusters). In the current study, we aim to investigate the temporal generalization of event file representations coded in the neurophysiological signal not only in the undecomposed EEG data but also in the decomposed C-, R-, and S-cluster data. To the best of our knowledge, this is the first study to combine the advantages of 2 contemporary methods: temporal decomposition and temporal generalization of neural time series. Due to the intermixed nature of the undecomposed EEG, we do not expect that event files cannot reliably be decoded from the signal. We hypothesize that event file representations are detectable in the C-, and not in the R-cluster data. Furthermore, we investigate the open question (see Opitz et al. 2020) whether even file representations are detectable in the S-cluster. If they prove to be detectable, we assume that event file representations have a larger activation in the C-, than in the S-cluster. If successful, the study provides the first in-depth analysis of representational stability of stimulus–response associations proposed by cognitive theory (i.e., TEC) reflected at a neurophysiological level.

## Materials and Methods

### Participants

For the behavioral analysis, a priori power analysis was conducted in G^*^Power (Faul et al. 2007). A minimum sample size of *N* = 34 was required to sufficiently power interaction effects given the alpha error probability is *P* < 0.005, and the repeated measures are not strongly correlated with each other (*r* < 0.25). Similar power analysis is not available for the MVPA. A sample of *N* = 40 (16 males and 24 females, age: *M* = 24.7, SD = 3.2 years) healthy young adults participated in the study. All participants had normal or corrected to normal vision. They did not report a history of psychiatric or neurological disorders or the use of centrally acting medication. All participants were undergraduate or graduate students and were financially reimbursed for their participation. All participants gave written informed consent prior to their participation in the study. The study was conducted in accordance with the Helsinki Declaration. The study was approved by the Ethics Committee of the TU Dresden.

### Task

**Figure 1:**
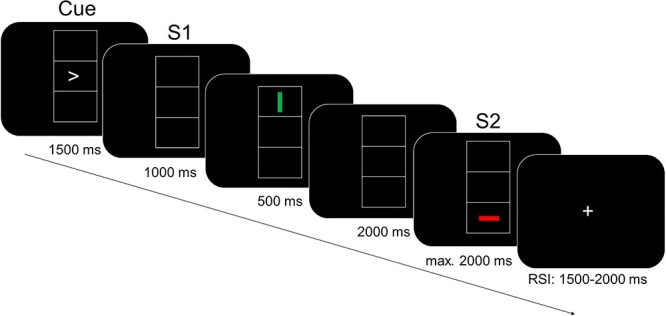
Schematic illustration of the event file coding task. The figure represents the order of the stimuli during a trial. The timing of the stimuli is described in the text.

Event file coding was examined by using an event file coding paradigm (Hommel 1998) also known as an S–R task (Colzato et al. 2006b). The task is depicted in Figure 1. The participants sat at a distance of 60 cm in front of a 17-inch CRT screen. During the experiments, the participants saw 3 vertically aligned boxes in the middle of a screen. Each box measured 2.8 × 2.2 cm. In the middle box, participants saw an arrowhead pointing to the left or right, representing the response cue. This was then followed by the consecutive presentation of 2 single-bar stimuli of 1.2 × 0.3 cm. Each of these bars could be oriented either vertically or horizontally (representing the task-relevant feature of orientation), could furthermore be either red or green (representing the task-irrelevant feature of color), and could be placed in either the top or the bottom box of the visual array (representing the task-irrelevant feature of location).These lines served as Stimulus 1 (S1) and Stimulus 2 (S2). In some trials none of these features were shared between S1 and S2 (zero feature overlap condition), other trials showed identical S1 and S2 (full feature overlap condition), and the remaining trials shared 1 or 2 features (partial feature overlap conditions: 1 feature and 2 feature overlap). Two responses (R1 and R2) had to be executed per trial by pressing the left or right control key on a computer keyboard with the corresponding index finger. Thus, in the task, 2 consecutive answers could require the same keystroke (response repetition) or 2 different ones (response alternation). The participants were informed that there would be no systematic relationship between S1 and R1 or between S1 and S2. Therefore, the task was designed to investigate automatic binding effects, i.e., the interactions between repetitions of stimulus features (overlapping of stimulus features) and responses. The timing of the experiment was as follows: In each trial, the cue initially appeared on the screen for 1500 ms. Participants were instructed not to react immediately to the cue, but to withhold their reaction until S1 was presented. After the response cue, a blank screen appeared for 1000 ms. Then S1 was displayed for 500 ms. Whenever S1 appeared, participants were expected to perform R1 (right press when the keyword was pointing to the right and vice versa). It is important to note that R1 was executed simultaneously with, but independently of, the orientation, color, or position of S1. Nevertheless, the proximity of S1 and R1 means that S1 became related to R1 (automatic binding). The display of S1 was followed by a blank screen for 2000 ms. Next, S2 was presented for 2000 ms or until a response was received. R2 required a response to the orientation of S2 (vertical vs. horizontal). The participants were instructed to press the left button when a horizontal line was shown and the right button when a vertical line was shown. If R1 was not correct, the trial was repeated once. The whole session consisted of 384 trials, which exceeded the maximum of 395 due to the repetition of the incorrect R1s. The number of trials was determined as a factorial combination of S2 characteristics, such as shape (2), ^*^ color (2), ^*^ location (2), the repetition versus change of shape (2), ^*^ the repetition vs. change of color (2), ^*^ the repetition versus change of location (2), ^*^ and the response (2). Each combination was repeated 3 times (Colzato et al. 2006b). During the intervals between the trials, which were jittered between 1500 and 2000 ms, a fixation cross was displayed in the middle of the screen.

Statistical analyses on the behavioral data were carried out with JASP. The mean accuracy (percentage of correct answers) and the medians of the RT data (for correct responses) were calculated for each participant and condition. Accuracy and RT data were analyzed in two-way repeated measures ANOVA with feature overlap (no, 1 feature overlap, 2 features overlap, and full overlap between S1 and S2 stimulus features) and response (repetition vs. switch) as within-subject factors. This approach is identical to earlier studies that investigated binding effects in event coding (Beste et al. 2016; Petruo et al. 2016). Here we report ${\eta}_{\mathrm{p}}^2$ effect size for ANOVA main effects and interactions. All post hoc tests were Bonferroni-corrected.

### E‌EG Data Acquisition and Processing

The EEG was recorded from 60 Ag/AgCl electrodes (EasyCap, Germany) in equidistant positions using a QuickAmp amplifier and the Brain Vision Recorder 1.2 software (Brain Products, Germany). The remaining EOG channels were disabled for recording. The ground and reference electrodes were placed at the coordinates θ = 58, φ = 78 and θ = 90, φ = 90, respectively. The sampling rate was 500 Hz. The data pre-processing was performed with the Brain Vision Analyzer 2 (Brain Products, Germany) and included the following steps: First, the data was down-sampled to 256 Hz and bandpass filtered (IIR filter: 0.5–40 Hz with an order of 8). The down-sampled data were re-referenced to an average reference. Then, a manual check of the data was performed to remove technical artifacts. The remaining artifacts with periodic effects such as blinking, eye movements, and pulse artifacts were removed by an independent component analysis (ICA, Infomax algorithm). Please note that the pre-processing was based on the established protocol of our lab, which has been extensively used for ERP, time–frequency, and connectivity research before (e.g., Dippel et al. 2017; Mückschel et al. 2017a, 2017b; Bensmann et al. 2019). Since MVPA is a relatively new approach to analyze EEG data, standards for pre-processing are not available yet (Carlson et al. 2019). However, the results were inspected to detect possible pre-processing-related artifacts, which were previously reported in MVPA literature (van Driel et al. 2019). Spurious, off-diagonal above-chance activities did not appear in the results of our analysis; therefore, we concluded that pre-processing was unlikely to create artifacts. The pre-processed data was segmented using epochs locked on the S2 (−1000 to 1000 ms). While the binding of the event file originally occurs after the establishment of the S1–R1 association, the binding has traditionally been studied with respect to retrieval, unbinding, and reconfiguration, which is required by the S2–R2 (Hommel 1998, 2004; Kühn et al. 2011). Only trials with correct R1 and R2 responses were included in the segmentation. Separate segments were created for all combinations of feature overlap levels (none, 1 feature overlaps, 2 features overlap, and full overlap between S1 and S2 stimulus features) and responses (repetition vs. alternation). On the segmented data, an automated artefact rejection procedure was applied in the time window of 1000 ms before and after the S2. This process discarded all segments with amplitudes higher than 150 μV, or lower than −150 μV, or activities lower than 0.5 μV over a time interval of at least 100 ms. Next, the segments were baseline corrected based on the mean activity from −200 to 0 ms (S2 onset). The pre-processed, segmented, and baseline-corrected data was used for temporal decomposition and for the MVPA of the undecomposed data. On the same data, event-related potential (ERP) analysis was performed based on previous studies which analyzed the P3 component in event file coding (Kleimaker et al. 2020; Takacs et al. 2020). The ERP results can be found in the Supplementary Materials.

### Residue Iteration Decomposition

RIDE postulates that different components with variable inter-component delays can be distinguished within ERPs (Ouyang et al. 2015a). Based on this assumption, RIDE decomposes the single-trial ERPs into different components with static or variable latencies. Depending on the timing and variability of these components, they can be linked to different stages of information processing. RIDE uses an iterative temporal decomposition, which has already been used earlier with robust results (Mückschel et al. 2017a; Ouyang et al. 2015b). The decomposition is applied separately for each electrode and is therefore sensitive to the channel-specific latency variability information (Ouyang et al. 2015a). In the current study, RIDE decomposition was performed according to established procedures (Ouyang et al. 2011; Verleger et al. 2014; Mückschel et al. 2017a) using the RIDE toolbox (for manual see http://cns.hkbu.edu.hk/RIDE.htm) in Matlab (Mathworks, Inc., Massachusetts, USA). We used latency information related to the stimulus and response onsets to derive the clusters S (stimulus) and R (response). The latency information of the C (central)-cluster is estimated and iteratively improved in each trial. RIDE requires predefined time windows to extract the waveforms for each cluster (Ouyang et al. 2011, 2015a). We applied the following intervals: for the S-cluster 200 ms before S2 and up to 700 ms after the S2 presentation; for the R-cluster 300 ms before and after R2; and for the C-cluster 150–800 ms after the S2 stimulus. These time windows correspond to previous studies of event file coding (Kleimaker et al. 2020; Takacs et al. 2020). For the details of selecting the time windows, please see Takacs et al. (2020). Using the provided markers, RIDE uses an iterative decomposition with an *L1*-norm minimization that produces median waveforms. To estimate the S-cluster, RIDE subtracts C and R from each study and adjusts the residual of all studies for the latency information of S. The result is the mean waveform for all time points in the S-cluster interval. The same procedure is used to derive clusters C and R. The whole process is iterated to improve the estimation of the components until they converge. For more details on the RIDE method, see Ouyang et al. (2011, 2015b)). After obtaining the RIDE clusters, we used them as input data for the MVPA process.

### Multivariate Pattern Analysis

We performed MVPA on the pre-processed and segmented, undecomposed EEG and also on the RIDE decomposed data using the ADAM toolbox (version 1.05, Fahrenfort et al. 2018) in Matlab (Mathworks). Prior to the MVPA, the EEG data was down-sampled offline to 55 Hz to facilitate temporal generalization (Fahrenfort et al. 2018). A linear discriminant classifier was trained and tested on each time point by using a 5-fold cross-validation. That is, the classifier was trained on 80% of the data and tested 20% of the data, repeating this process until all data chunks have been tested. The Area under the ROC Curve (AUC) was used as a measure of classification accuracy. Larger area indicates more accurate classification performance (Fahrenfort et al. 2018). The final performance metric was computed by creating the average of test folds. Two categories were used to train the classifier: zero overlap with response alternation and full feature overlap with response alternation (for a similar approach, see Akçay and Hazeltine 2007). These 2 categories represent similar levels of response selection (i.e., switching from the previously activated response): With zero feature overlap, the original S–R relation remains intact, while with full feature overlap the S–R association needs to be unbound and reconfigured. That is, when a new response is required (response alternation) and the stimulus is also new (zero overlap with S1), the original binding between S1 and R1 does not need to be retrieved and used. However, when the S2 overlaps with S1, the original association between S1 and R1 is reactivated. Since the required response is new (response alternation), this original binding needs to be modified for successful action control. That is, the difference between zero overlap with response alternation and full feature overlap with response alternation should necessarily provide information on the representation of event files. In the former case, event files do not play a role, while in the latter, the event files are retrieved and modulated. For the sake of completion, in the Supplementary Material, we also report the classification results between zero overlap with response repetition and full feature overlap with response repetition conditions. In case of unbalanced trial numbers in the categories, the majority class has been down-sampled to avoid skewed classification (Fahrenfort et al. 2018). All electrodes were included in the analysis. The EEG amplitudes at individual electrode channels were used as classification features, creating 60 features in both stimulus classes. A backward decoding model (BDM) (Fahrenfort et al. 2018) was used for training and computing metric on testing. Next, temporal generalization matrices were calculated by using cross-classification across time. This process is looking for clusters of contiguous time samples that remain significant after random permutation. In this step, the stability of the observed pattern (undecomposed EEG, or decomposed C-, R-, and S-cluster activity) was evaluated over time by training the model in one time point and testing its discrimination performance in the remaining time points. Cross-classification was repeated for every time point. As a result, classification performance above-chance level outside the diagonal axis indicates sustained neural activity. Additionally, topographical maps were created based on classifier weights for the individual electrode channels. Statistical analyses for the MVPA, that is, group statistics and multiple correction, were performed in ADAM (Fahrenfort et al. 2018). Two-sided *t*-tests against chance level (AUC = 0.05) were performed for each time sample across subjects. Cluster-based permutation was used as correction for multiple comparisons. Clusters were treated as contiguously significant *t*-tests. The sum of the *t*-values in a cluster was used to determine cluster size. This procedure was repeated 1000 times. For each participant and each repetition, AUC was set to chance level before computing the *t*-test. Null distribution of cluster sizes under random permutation was calculated against the observed cluster sizes, and this comparison was used to calculate the *P*-values of clusters (Fahrenfort et al. 2018).

**Figure 2:**
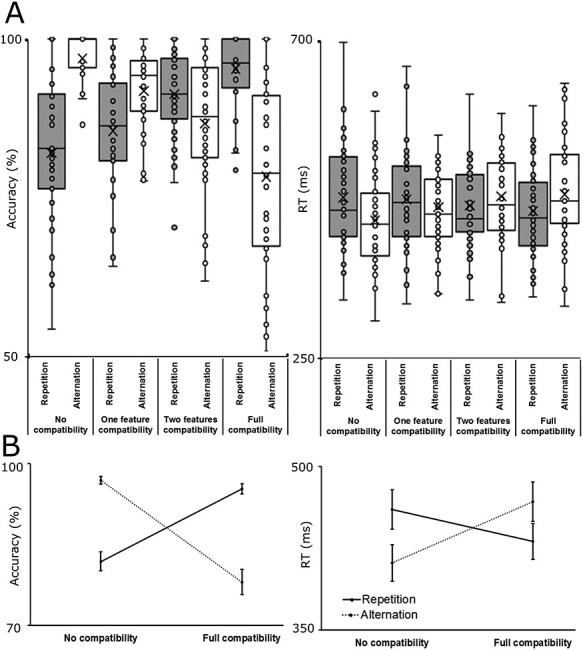
(*A*) Accuracy results across feature overlap and response-type conditions. The percentage of correct trials is shown as a function of overlapping features for repeated and alternated responses. Repeated responses are indicated by solid lines; alternated responses are indicated by dotted lines. Error bars denote standard error of mean. (*B*) Reaction times across feature overlap and response-type conditions. The mean RT is shown as a function of overlapping features for repeated and alternated responses. Repeated responses are indicated by solid lines; alternated responses are indicated by dotted lines. Error bars denote standard error of mean.

## Results

### Behavioral Data

The response accuracy data is shown in Figure 2. The ANOVA with the factors feature overlap and response type (i.e., repetition/alternation) on the accuracy data showed that the main effect of feature overlap was significant (*F*(3,117) = 4.11, Ɛ = 0.739, *P* = 0.016, ${\eta}_{\mathrm{p}}^2$ = 0.095). Responses were more accurate in the full feature overlap (86.7% *±* 1.3) than in the 2 feature overlap conditions (88.9% *±* 1.2, *P* = 0.038). No other pairwise differences were significant (*P* > 0.118). The main effect of response type (*F*(1,39) = 0.01, *P* = 0.916, ${\eta}_{\mathrm{p}}^2$ = 0.001) was not significant. Importantly, however, the feature overlap by response interaction was significant (*F*(3,117) = 69.44, Ɛ = 0.581, *P* < 0.001, ${\eta}_{\mathrm{p}}^2$ = 0.640). When responses had to be repeated, accuracy increased from the zero overlap (81.9% *±* 1.8) to the 1 feature overlap (85.4% *±* 1.5, *P* = 0.045), to the 2 features overlap (91.2% *±* 1.1, *P* < 0.001), and the full overlap (95.3% *±* 0.9, *P* < 0.001) conditions. This reflects the usually found partial repetition benefit effect reported in literature (Hommel et al. 2001a; Colzato et al. 2006a, 2006b, 2013). Accuracy was better in the full feature overlap than in the 2 features overlap (*P* = 0.005) and in the 1 feature overlap conditions (*P* < 0.001). Finally, accuracy was higher in the 2 features overlap than in the 1 feature overlap condition (*P* < 0.001). When responses had to be alternated, accuracy decreased from the zero overlap (96.9% *±* 0.6) to the 1 feature overlap (91.8% *±* 1.0, *P* < 0.001), to the 2 features overlap (86.6% *±* 1.6, *P* < 0.001), and the full overlap (78.1% *±* 2.4, *P* < 0.001) conditions. This reflects the usually found partial repetition costs effect reported in literature (Hommel et al. 2001a; Colzato et al. 2006a, 2006b, 2013). Additionally, accuracy was worse in the full feature overlap than in the 2 features overlap (*P* < 0.001) and in the 1 feature overlap conditions (*P* < 0.001). Finally, accuracy was lower in the 2 features overlap than in the 1 feature overlap condition (*P* < 0.001).

The ANOVA with the factors feature overlap and response type (i.e., repetition/alternation) on the reaction time (RT) data showed that the main effect of feature overlap (*F*(3,117) = 3.89, Ɛ = 0.681, *P* = 0.024, ${\eta}_{\mathrm{p}}^2$ = 0.091) was significant. Responses were faster in the no feature overlap (457.4 ms *±* 11.1) than in the 2 features overlap condition (468.4 ms *±* 11.1, *P* = 0.001). No other pairwise comparisons were significant (*P* > 0.064). The main effect of response type (*F*(1,39) = 0.42, *P* = 0.522, ${\eta}_{\mathrm{p}}^2$ = 0.011) was not significant. However, again, the feature overlap by response interaction was significant (*F*(3,117) = 33.24, *P* < 0.001, ${\eta}_{\mathrm{p}}^2$ = 0.460). When responses had to be repeated, RT decreased from the no feature overlap (473.7 ms *±* 11.8) to the full feature overlap condition (454.3 ms *±* 10.9, *P* = 0.007). Furthermore, participants were slower in the 1 feature overlap (471.0 ms *±* 11.0) than in the 2 features overlap (462.2 ms *±* 10.5, *P* = 0.047) and in the full feature overlap conditions (454.3 ms *±* 10.9, *P* = 0.003). When responses had to be alternated, RT increased from the no feature overlap (441.2 ms *±* 11.0) to the 1 feature overlap (460.1 ms *±* 10.6, *P* = 0.001), 2 features overlap (475.7 ms *±* 11.6, *P* < 0.001), and full overlap conditions (478.4 ms *±* 11.8, *P* < 0.001). Furthermore, participants were faster in the 1 feature overlap than in the 2 features overlap (*P* < 0.001) and in the full feature overlap conditions (*P* = 0.020). No other pairwise comparisons were significant (*p* > 0.067).

### Neurophysiological Data

**Figure 3:**
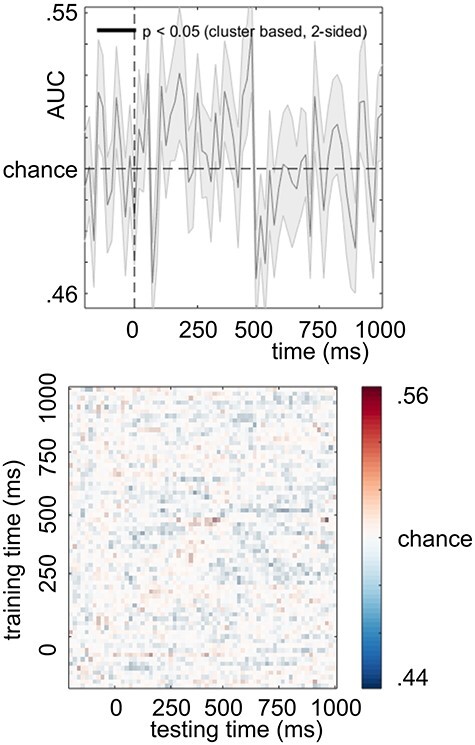
Decoding accuracy and temporal generalization matrix for the undecomposed EEG. The top panel shows the classification performance across time between no feature overlap with response alternation and full feature overlap with response alternation for the undecomposed EEG. The bottom panel shows the result of the temporal generalization.

After replicating the main behavioral effects known in the S–R task (Hommel et al. 2001a; Colzato et al. 2006a, 2006b, 2013), we limited the neurophysiological analysis to the focus of the study. That is, the difference between 2 conditions: zero overlap with response alternation and full feature overlap with response alternation (see Multivariate Pattern Analysis). First, we present the decoding accuracy results to report the performance of the classification. Next, we present the temporal generalization, that is, the stability of the event file representations. We provide this information separately for the undecomposed EEG and for the decomposed C-, R-, and S-cluster data. The decoding accuracy did not reach significance for the undecomposed EEG data (*p* > 0.05). The left panel of Figure 3 shows that decoding accuracy was consequently around chance level.

**Figure 4:**
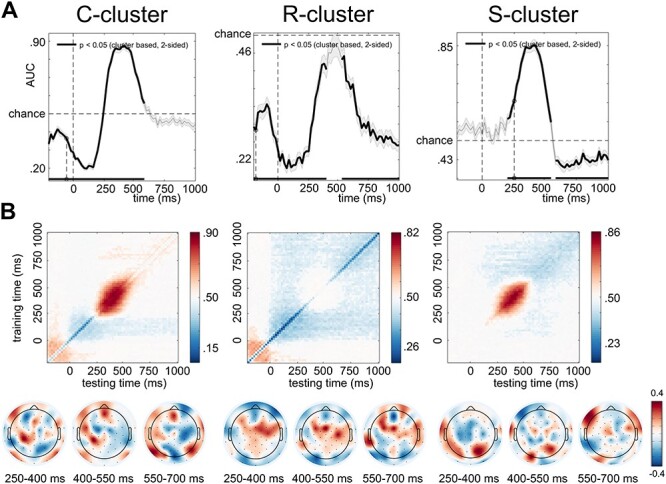
Decoding accuracy and temporal generalization matrix for the C-, R-, and S-cluster data. (*A*) Decoding accuracy for the 3 RIDE clusters. Significant time windows (*P* < 0.05, after cluster-based permutation) are indicated by thicker lines. (*B*) Temporal generalization matrices and maps of forward transformation weights of the decomposed EEG data. Significant samples are indicated by saturated colors. Unsaturated colors represent *P*-values below the multiple-comparison corrected threshold.

Therefore, classes of no feature overlap with response alternation and full feature overlap with response alternation could not be reliably differentiated. Consequently, the temporal generalization matrix (depicted on the right panel of Figure 3) shows that there is no significant above- or below-chance activity detected in the neural signal. The plot would indicate that the success of the classifier when trained on the data (*y*-axis) generalizes to other data points (*x*-axis). The scattered pattern depicted in Figure 3 shows that event file coding processes were not detectable in the undecomposed neural signal. The decoding accuracy (AUC) results for the C-, R-, and S-clusters are shown in Figure 4A.

In the C-cluster, significant differences (*p* < 0.05) were found between the classes of no feature overlap with response alternation and full feature overlap with response alternation from the onset of S2 (i.e., time point zero) to 600 ms after the onset of S2. In the R-cluster, significant below-chance difference (*p* < 0.05) was found between the 2 classes from the stimulus onset to 300 ms and from 550 to 1000 ms. In the S-cluster, significant above-chance difference (*p* < 0.05) was found between the 2 classes from 240 to 550 ms after S2 onset. Additionally, significant below-chance difference (*p* < 0.05) was found between the 2 classes from 560 to 1000 ms. Since in all 3 decomposed datasets the classification was successful, in the next step, we calculated the temporal generalization matrices to investigate the stability of the representations. The temporal generalization results for the C-, R-, and S-clusters are shown in Figure 4B. In the C-cluster, above-chance activity was detected between 250 and 600 ms after the onset of S2. Additionally, a smaller cluster was detected significantly below-chance level between 50 and 240 ms. In the R-cluster, a transient, quadratic cluster was detected significantly below-chance level between stimulus onset and the end of the investigated segment (1000 ms). Importantly, the activation was not homogenous: classification performance was nonsignificant for a jittered diagonal pattern between 300 and 600 ms. That is, the main above-chance activation in the C-cluster was characterized by an unsuccessful classification in the same time window in the R-cluster. This mirror-reversed pattern was also observable in the S-cluster. Specifically, in the S-cluster, above-chance activity was detected between 250 and 550 ms after the onset of the stimulus, similar to the main activation of the C-cluster. It was followed by a significant below-chance activity until the end of the segment. In sum, decoding accuracy showed successful classification performance in all 3 temporally decomposed clusters. The temporal generalization matrices indicated different sensitivities to event file representations. In the C- and S-clusters, significant above-chance activities showed stable event file representations between 250–600 ms and 250–550 ms, respectively. In contrast, only below-chance activation was detected in the R-cluster.

## Discussion

In the current study, we investigated how the representational content of event files, a central element in the theory of event coding (TEC) (Hommel 2011), is coded at the neurophysiological level. Until now, research on neural correlates of event file coding has focused on functional neuroanatomical regions involved in event coding or the time course in cognitive subprocesses (ERP-correlates) involved. Critically, the representational content of the event files has been rarely examined at the neurophysiological level (Kikumoto and Mayr 2019), although it is the strength of representations in an event file that is of most theoretical importance from a cognitive perspective and underlies the behavioral signatures of even file coding (Hommel 2009). Importantly, the TEC does not specify how event files are coded on a neurophysiological level. The current study closes this gap.

Regarding that behavioral data, the analysis replicated the well-known effects of event file binding (Hommel 2004; Colzato et al. 2006a). Namely, participants were the most accurate and the fastest when features of consecutive stimuli were highly overlapping and responses needed to be repeated (partial repetition benefit). However, participants’ speed and accuracy deteriorated when their response had to be altered and there was a high stimulus feature overlap (partial repetition cost). Thus, participants’ behavioral performance was modulated by the formation of event files: pre-established bindings led to response facilitation, while unbinding caused an inhibitory effect (Hommel 2004; Colzato et al. 2006a). Thus, event files are crucial elements to understand the cognition of action control (Frings et al. 2020). The decoding of the event files’ representational content, using MVPA, revealed several theoretical important findings:

Decoding of event file representations was around chance level when the classification was applied to undecomposed EEG. It appears that in the EEG, an intermix of different signals related to parallel processing and coding of different aspects of information during response selection (Mückschel et al. 2017a) presents a general challenge in analyzing event file coding in neurophysiological data. Crucially, this makes sense from the perspective of TEC. Namely, the TEC assumes a complex architecture, which involves the existence of action, object, and event files, all of them connected and represented in parallel in a network of feature codes (Hommel et al. 2001a, 2001b; Hommel 2019). However, in the undecomposed neural signal, the elements of TEC are not necessarily distinguishable from each other (Folstein and Van Petten 2008; Kleimaker et al. 2020; Opitz et al. 2020; Takacs et al. 2020). Several findings have suggested that there are different coding levels intermingled in the EEG signal that play specific roles during response selection and cognitive control (Mückschel et al. 2017a, 2017b; Chmielewski et al. 2018). Separating these intermingled coding levels requires dedicated signal processing methods, such as temporal decomposition (Ouyang et al. 2011, 2015a). While other methods, such as spatial decomposition, are also available, recent studies of event file coding and response selection (Verleger et al. 2014; Mückschel et al. 2017a; Opitz et al. 2020; Takacs et al. 2020) proved that temporal decomposition is useful to obtain reliable and meaningful components from the mixed signal. Corroborating this, the results from the MVPA analysis based on the RIDE-decomposed EEG data yielded successful decoding of event files. Crucially, the 3 RIDE clusters (S, C, and R) are characterized by 3 distinguishable, and partly opposed, temporal generalization patterns. These differences highlight how response- and stimulus-related and translational aspects contribute to the representations of event files.

Specifically, the C-cluster showed evidence for a sustained and temporally sustained neural activity during event file coding. As shown in Figure 4, the sustained activation was reflected in a diagonal matrix pattern between 250 and 600 ms after the second stimulus presentation, that is, when the original S–R association needed to be retrieved and reconfigured. The above-chance activation indicates that event files were represented in the brain in a time window corresponding to both the N2 and P3 ERP components. Previous ERP research proposed that N2 and P3 can be both implicated in event file coding (Petruo et al. 2016, 2018; Kleimaker et al. 2020; Opitz et al. 2020; Takacs et al. 2020), which is in line with findings suggesting that modulations in the N2/P3 time window are generally found during response selection processes (Ullsperger et al. 2014). The current findings of a temporal jittered and a smoothed activation pattern both horizontally and vertically indicate that neural activity gradually evolves during event file coding, suggesting that there is no categorical nature of event files. This potentially indicates that event files are activated in a gradual manner and that it takes about 350 ms to open, operate, and close event files. As the activation spreads in the network of feature codes, the event file slowly becomes more stable, and when it is not needed anymore, the activation gradually fades away. This relative instability of event file representation is important, because only a certain level of instability of task-related representations facilitates the generalizability of these representations to other domains (Robertson 2018). Generalization requires common features across representational contents, which is suggested to be only possible when the episodic trace for features is partially instable (Robertson 2018). Event files have been considered to reflect episodic (memory) traces (Hommel 2009; Frings et al. 2020), and it has also proposed that generalizability through feature codes is an important capability of event files (Hommel et al. 2001a; Kikumoto and Mayr 2019). The current study is the first to show these aspects on a level of temporal generalizability and suggest that event file representations are coded by a specific aspect in the neurophysiological signal from 250 to 600 ms. This time window corresponds only partially to a recent study’s finding (Kikumoto and Mayr 2019), in which conjunctive representations of stimulus–response associations arose right after the stimulus presentation and lasted until the response execution. This difference can be a result of the choice of methods. In the study of Kikumoto and Mayr, representational similarity analysis was used based on time–frequency decomposed data. The 5 frequency bands and the 20 included electrodes resulted in 100 features, which might provide a lower precision than the temporal generalizability of segmented EEG data. The finding in the C-cluster, which has been suggested to reflect stimulus–response transition/association processes (Verleger et al. 2014; Ouyang et al. 2017; Opitz et al. 2020; Takacs et al. 2020), is well in line with the theoretical framing of event files on a cognitive level (Hommel 2009). Yet, the main activation between 250 and 600 ms was embedded in above- and below-chance activations. The exact functional meaning of such recurring neural activity pattern remains an open question (King and Dehaene 2014). For instance, it has been suggested that neuronal assemblies firing at stimulus onset fall below baseline firing rates after the stimulus onset (Carlson et al. 2011). In the current study, the time intervals of the early activities do not correspond to stimulus onset-offset. Rather, a pre-stimulus above-chance activity led to a transient below-chance performance after the stimulus presentation. Importantly, in case of a C-cluster, we should also not expect strictly stimulus-driven processes to be present in the signal, as those are decomposed in the S-cluster (Ouyang et al. 2011, 2015a; Mückschel et al. 2017a). However, in the C-cluster, response selection mechanisms related to an anticipation of the response, and then the suppression of it when the response had to be alternated, could potentially be reflected by the early reversing components. This has direct relevance to the findings in the R-cluster:

In the R-cluster, the temporal generalization showed a ramping activity starting from the stimulus onset. Like the C-cluster, this was preceded by an above-chance activation just before the stimulus onset. The change between above- and below-chance activities probably reflects that neurons active during the above-chance time window became inactive afterward (King and Dehaene 2014). This pattern might be interpreted in that a previous motor representation is active but becomes suppressed after the S2 presentation. Since the R-cluster reflects motor execution-related processes (Ouyang et al. 2011, 2015a; Mückschel et al. 2017a), this further strengthens the possibility that response selection mechanisms are behind the early above-chance activation. As we only included trials with response alternation to the analysis, the previously primed response (R1) and the anticipations triggered by it had to be suppressed (for an analysis with response repetition trials, see Supplementary Material). This was potentially reflected by the below-chance activity following the stimulus presentation. While this transient change was only briefly presented in the C-cluster data, it was dominant in the R-cluster. Importantly, the below-chance activation had a unique shape: The ramping, rectangular activation pattern had a jittered diagonal area of nonsignificant activation. Curiously, this nonsignificant activation was characterized by a time window of 300–600 ms, roughly corresponding to the main activation pattern in the C-cluster data. This might suggest that event file representation as presented in the C-cluster data is independent from R-cluster activities. That is, event files are cognitive representations, defined by S–R translation processes, not including a representation of the motor execution process. This is in line with the notion that event files cannot be explained only by response-related mechanisms (Colzato et al. 2006a; Hommel 2019; Opitz et al. 2020). The finding of an opposed pattern of representations in the C-cluster and the R-cluster also explains why no reliable MVPA result was obtained on the undecomposed EEG data.

In contrast to the R-cluster’s profile, the S-cluster showed a pattern like the C-cluster activation. Namely, representations were detectable between 250 and 550 ms after the stimulus onset, corresponding to the time window of the main above-chance activity in the C-cluster. Moreover, the S- and C-cluster activations showed a similar temporal generalization pattern. However, the S-cluster activation was smaller both vertically and horizontally than the C-cluster’s main activation. The activation in the S-cluster potentially indicates that stimulus-level representations contribute to the event files. However, as the activation had a smaller area both horizontally and vertically, the stimulus level may be represented to a lesser extent than S–R translation processes. This interpretation is in line with previous ERP results (Kleimaker et al. 2020; Opitz et al. 2020; Takacs et al. 2020), which showed that event file coding is predominantly related to C-cluster activation.

Overall, the 3 temporally decomposed clusters showed both similarities and dissimilarities with each other that delineate propositions of TEC at the neurophysiological level: The TEC proposes that perceptual contents and action plans are equally represented by integrated networks of feature codes (Hommel et al. 2001a, 2001b). These so-called event files refer to the features of the represented event, that is, the neural firing pattern should reflect the event itself, and not the stimulus or motor codes separately (Hommel et al. 2001a, 2001b; Hommel 2019). In turn, stimulus and motor codes are not similar to each other. The current findings partially corroborate with these predictions. Indeed, the stimulus and motor codes indicated by the S- and R-cluster data were non-similar. However, the similarity of the main activation in the C- and S-clusters in comparison to the R-cluster would suggest that event files rely more on the stimulus codes than on the motor codes. It is important to note that the current study does not propose to eliminate the role of action representations from event files. Rather, it draws a picture that event files at the neural coding level have more in common with the perceptual aspect than with the motor code. The opposed relationship between the C- and R-cluster activations may suggest that event files and motor response specifications (action files) are intertwined differently than event files and stimulus feature specifications (object files). How the code sharing between file types takes place on a neurophysiological level remains an open question for future studies. Furthermore, the current study’s design is not suitable to distinguish 2 core aspects of action control: the initial binding of features and the retrieval of them (Frings et al. 2020). Thus, we also could not identify of how event file representations contribute to these different stages of information processing. This should be addressed in future research with different paradigms.

## Conclusion

Altogether, the current results provide first-hand evidence of the stability of event file codes at the neurophysiological level. This was achieved concatenating temporal EEG signal decomposition methods and MVPA. We show that undecomposed neurophysiological data is unsuitable to decode event file representations, because different aspects of information coded in the neurophysiological signal reveal distinct and partly opposed dynamics in the representational content. This is confirmed by applying MVPA to temporal decomposed EEG data. After intermingled coding levels have been separated, a reliable examination of the event file’s representational content is its stability over time. This provides important insights suggesting that the temporal stability of event file processes shows distinct profiles depending on the aspect of information being processed (i.e., stimulus-related, motor response-related, or processes linking stimulus evaluation and responding [stimulus–response bindings]). We show that representations, particularly of stimulus–response bindings, are activated and decay in a gradual manner. The relative instability during activation and decay could indicate the generalizability of event files, that is, event files can potentially influence information processing outside of their original context, as well. Moreover, event file representations resemble distributed rather than a focal neural activity. Especially representations of stimulus–response associations, as well as stimulus-related representations, are coded and reveal temporal stability. Purely, motor-related representations are not found in neurophysiological signals during event coding.

## Notes

***Conflict of Interest***: None declared.

## Funding

Deutsche Forschungsgemeinschaft (DFG) (grant FOR 2698).

## Supplementary Material

Supplementary_Materials_tgaa016
