# Supplementary material for: Decoding Stimulus–Response Representations and Their Stability Using EEG-Based Multivariate Pattern Analysis
Source: Cereb Cortex Commun. 2020 May 7;1(1):tgaa016. doi: 10.1093/texcom/tgaa016 (PMC8152870; doi:10.1093/texcom/tgaa016)
Supplement: Supplementary_Materials_tgaa016 [file supplementary_materials_tgaa016.docx]

**Supplementary Materials**

- 1. **Supplementary Results**
  2. **P3 component**

The P3 component for event file coding was analysed according to previous publications (Kleimaker et al., 2020; Takacs et al., 2020). The ERP data is presented in Supplementary Figure 1. To analyse the stimulus-locked P3 component, we selected the electrode Cz with a time window of 400 to 700 ms after the presentation of S2, based on similar studies which used the same S-R paradigm (Kleimaker et al., 2020; Takacs et al., 2020). Within this time interval, the mean amplitude was quantified and extracted at the single subject level. The compatibility by response ANOVA on the mean amplitude of the P3 showed that the main effects of compatibility (*F*(1,37) = 2.85, *p* = .100, η^2^ = .072, *BF*_10_ = 0.51), and response type (*F*(1,37) = 2.51, *p* = .121, η^2^ = .064, *BF*_10_ = 0.48) were not significant. The compatibility by response interaction was marginally significant (*F*(1,37) = 3.83, *p* = .058, η^2^ = .094, *BF*_10_ = 1.08).


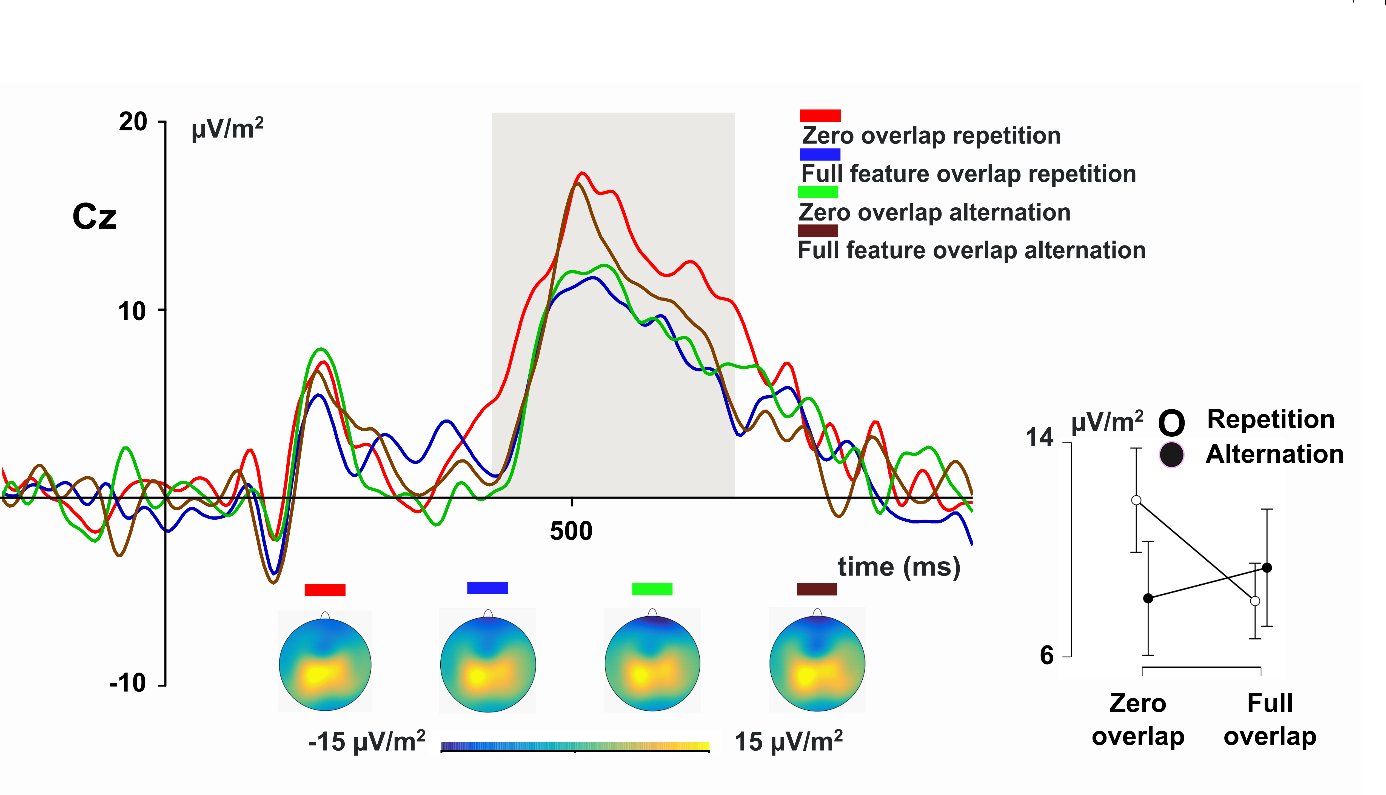


**Supplementary Figure 1.** ERP results at electrode Cz. The P3 component is shown across four conditions: zero overlap repetition (red), full feature overlap repetition (blue), zero overlap alternation (green), and full feature overlap alternation (brown). Time point zero denotes the stimulus presentation. The shaded area represents the analysed time window. The scalp topography plots show the distribution of the mean activity across the four conditions for the time window of the P3. The line chart on the right side depicts the mean amplitude of the P3 in the analysed time window across the four conditions.

- 1. **Multivariate pattern analysis**

The MVPA approach was identical to the one reported in the main text (2.4. Multivariate Pattern Analysis) with the exception of the included experimental conditions. Two categories were used to train the classifier: zero overlap with response repetition, and full feature overlap with response repetition. Decoding accuracy and temporal generalization results for the undecomposed EEG data are depicted in Supplementary Figure 2. Decoding accuracy and temporal generalization results for the RIDE decomposed C-, R-, and S-cluster data are depicted in Supplementary Figure 3.


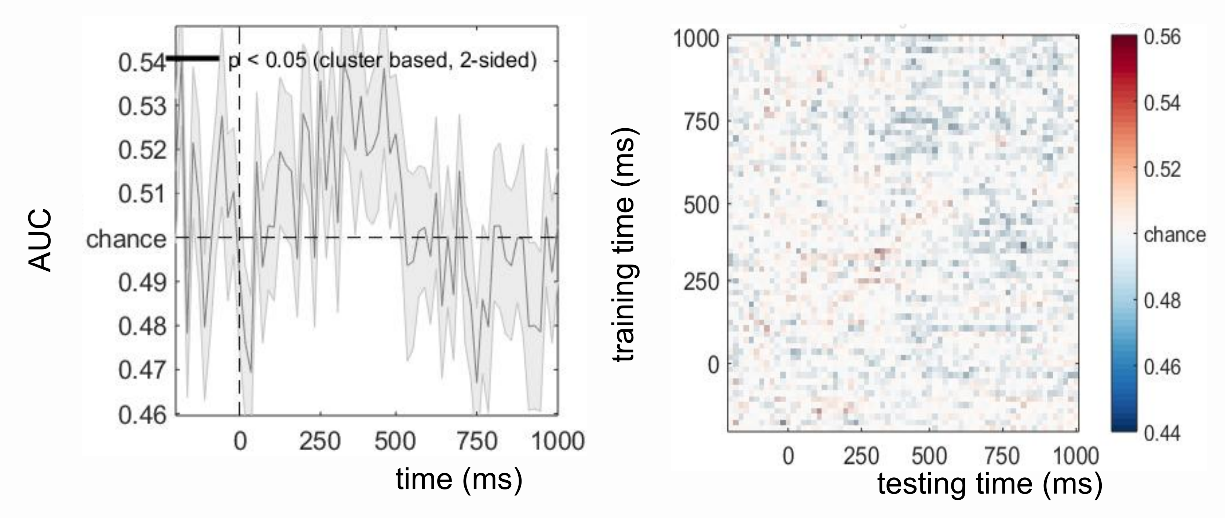


**Supplementary Figure 2.** Decoding accuracy and temporal generalization matrix for the undecomposed EEG. The left panel shows the classification performance across time between no feature overlap with response repetition and full feature overlap with response repetition for the undecomposed EEG. The right panel shows the result of the temporal generalization.

**
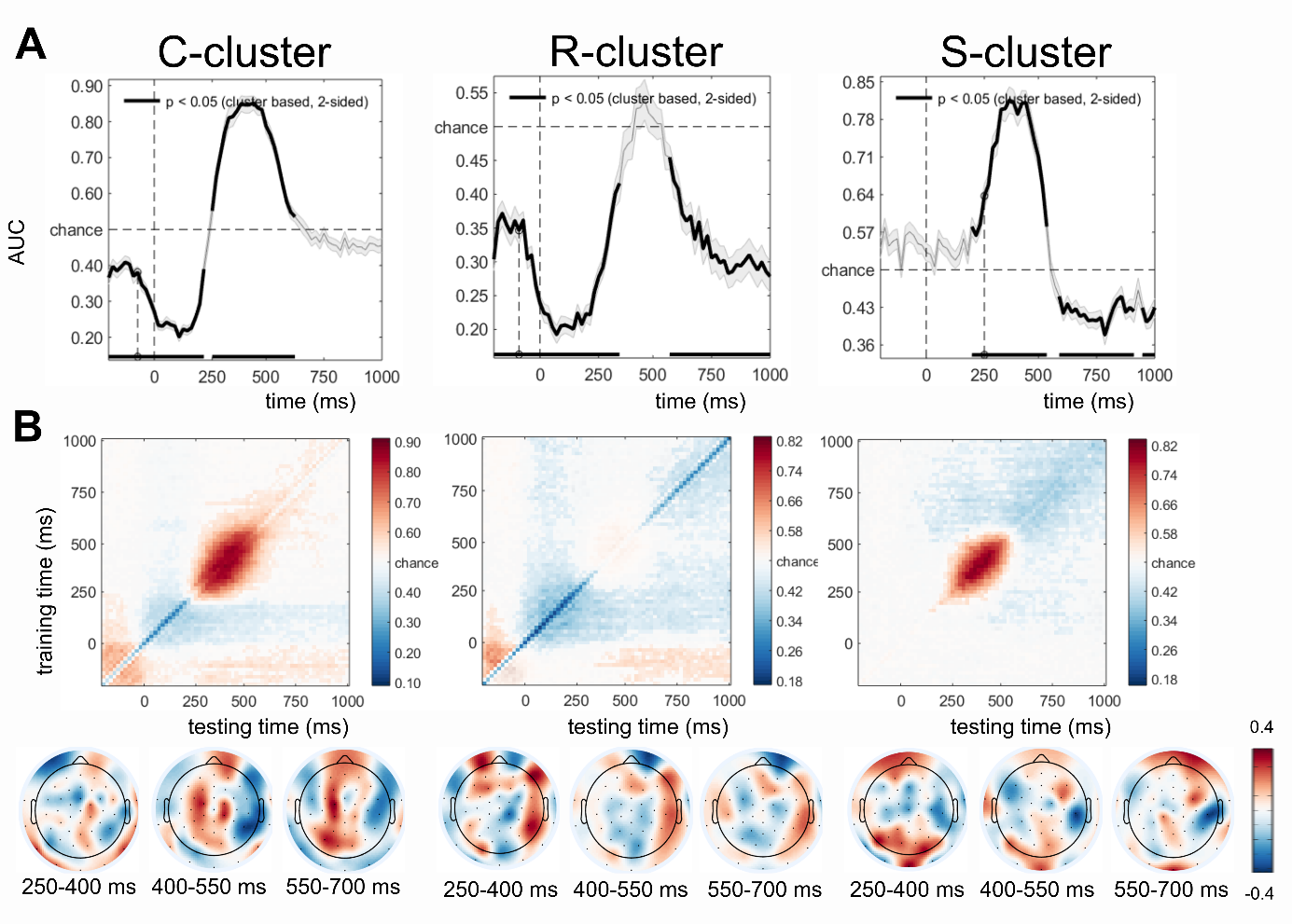
**

**Supplementary Figure 3.** Decoding accuracy and temporal generalization matrix for the C-, R-, and S-cluster data. A: Decoding accuracy for the three RIDE clusters. Significant time windows (*p* < .05, after cluster-based permutation) are indicated by thicker lines. B: Temporal generalization matrices and maps of forward transformation weights of the decomposed EEG data. Significant samples are indicated by saturated colours. Unsaturated colours represent *p*-values below the multiple-comparison corrected threshold.
